# A153 STRATEGIES TO DISTINGUISH PERIANAL FISTULAS RELATED TO CROHN’S DISEASE FROM CRYPTOGLANDULAR DISEASE: SYSTEMATIC REVIEW WITH META-ANALYSIS

**DOI:** 10.1093/jcag/gwab049.152

**Published:** 2022-02-21

**Authors:** K Chin Koon Siw, J Engel, S Visva, R Mallick, A Hart, A de Buck van Overstraeten, J McCurdy

**Affiliations:** 1 Division of Gastroenterology and Hepatology, Department of Medicine, University of Ottawa, Ottawa, ON, Canada; 2 University of Ottawa Faculty of Medicine, Ottawa, ON, Canada; 3 The Ottawa Hospital Research Institute, Ottawa, ON, Canada; 4 IBD Unit, St Mark’s Hospital, Harrow, London, United Kingdom; 5 Department of Surgery, Mount Sinai Hospital, University of Toronto, Toronto, ON, Canada

## Abstract

**Background:**

Differentiating between perianal fistulas related to cryptoglandular disease (CGD) and Crohn’s disease (CD) is essential to guide disease specific management.

**Aims:**

We aimed to assess the ability of diagnostic strategies to differentiate between CD from CGD in patients with perianal fistulas.

**Methods:**

We performed a diagnostic accuracy systematic review and meta-analysis. Electronic databases (MEDLINE, Embase, Web of Science, CENTRAL) were systematically searched from inception through February 2021 for studies that assessed a diagnostic test’s ability to distinguish fistula types. We calculated weighted summary estimates with 95% confidence intervals for sensitivity and specificity by bivariate analysis, using fixed effects models when data was available from two or more studies. The QUADAS tool was used to assess study quality.

**Results:**

Twenty-one studies were identified and included: clinical symptoms (2 studies; n = 154 patients), MRI characteristics (3 studies; n = 296 patients), ultrasound characteristics (7 studies; n = 1003 patients), video capsule endoscopy (2 studies; n = 44 patients), fecal calprotectin (1 study; n = 56 patients) and various biomarkers (8 studies; n = 440 patients). MRI and ultrasound characteristics had the most robust data. Rectal inflammation, multiple-branched fistula tracts and abscesses on pelvic MRI, and the Crohn’s Ultrasound Fistula Sign, fistula debris and bifurcated fistulas on pelvic ultrasonography had high specificity (range, 80–95% vs. 89–96%) but poor sensitivity (range, 17–37% vs. 31–63%), respectively. Fourteen of twenty-one studies had risk of bias on at least one of the QUADAS domains.

**Conclusions:**

Limited high-quality evidence suggest that imaging characteristics may help discriminate CD from CGD in patients with perianal fistulas. Larger, prospective studies are needed to confirm these findings and to evaluate if combining multiple diagnostic tests can improve diagnostic sensitivity.

**Funding Agencies:**

None

